# Ameloblastoma on the Maxillary Sinus: Cause of Unilateral Nasal Obstruction

**DOI:** 10.7759/cureus.6563

**Published:** 2020-01-04

**Authors:** Panagiota Kosmidou, Stavros Angelis, Eleni Papagianni, Iro Kokkevi, Dimitrios Filippou

**Affiliations:** 1 Otolaryngology - Head and Neck Surgery, Evangelismos General Hospital, Athens, GRC; 2 Orthopaedics, General Hospital Hellenic Red Cross Korgialenio Benakio, Athens, GRC; 3 Otolaryngology - Head and Neck Surgery, Army Share Fund Hospital (NIMTS), Athens, GRC; 4 Surgery, National and Kapodistrian University of Athens School of Medicine, Athens, GRC

**Keywords:** ameloblastoma, adamantinoma, odontogenic neoplasms, teeth

## Abstract

Ameloblastoma is a rare, benign (99%) or malignant (1%) tumour, which has derived from dental mesenchyme. We present a case of a patient mainly complaining about obstruction on the right nasal cavity. Endoscopy and computed tomography revealed a soft tissue mass occupying the maxillary sinus to the middle meatus causing complete obstruction of the right nasal cavity. Endoscopic en block removal of the lesion and biopsy confirmed follicular ameloblastoma. Literature review confirms the extremely rare frequency of ameloblastoma. The aim of the present study is to expand on our knowledge of a rare pathological entity that can frequently be misdiagnosed.

## Introduction

Ameloblastoma has also been called adamantinoma in the past [1]. It is mostly a benign tumour (99%), while malignant ameloblastomas are extremely rare (1%). Many experts do not accept their existence [1-4]. However, malignant ameloblastoma is described as a process that gives metastases to the lungs or to the epithelial lymph nodes. Histologically it falls under the odontogenic neoplasms from epithelial tissue [2]. Ameloblastoma is the most common neoplastic proliferation originating from dental tissue [1]. It is recognized in one out of nine (11%-13%) cases of tumours that are derived from the teeth, while it constitutes only 1% of the total solid and cystic processes of the jaws [3].

Four out of five cases are found on the mandible. Cases of ameloblastomas with upper jaw localization are rare (20%) and often confused with basal cell epitheliums [3,5]. Basal cell type comprises about 1%, but it often displays malignant behaviour [5]. Ameloblastoma is usually of late diagnosis because of its poor symptoms and low prevalence [4]. It is known for growing at the expense of surrounding tissues producing only blurry symptoms [3].

We present a case of a patient mainly complaining about obstruction on the right nasal cavity. Examination and diagnostic imaging revealed a soft tissue mass lesion occupying the maxillary sinus to the middle meatus. This resulted in complete obstruction of the right nasal cavity. Removal of the lesion and biopsy confirmed follicular ameloblastoma. Atypical localization and lack of basal cell type behaviour make this case infrequent. The aim of the present study is to expand on our knowledge of a rare pathological entity that can frequently be misdiagnosed.

## Case presentation

**Figure 1:**
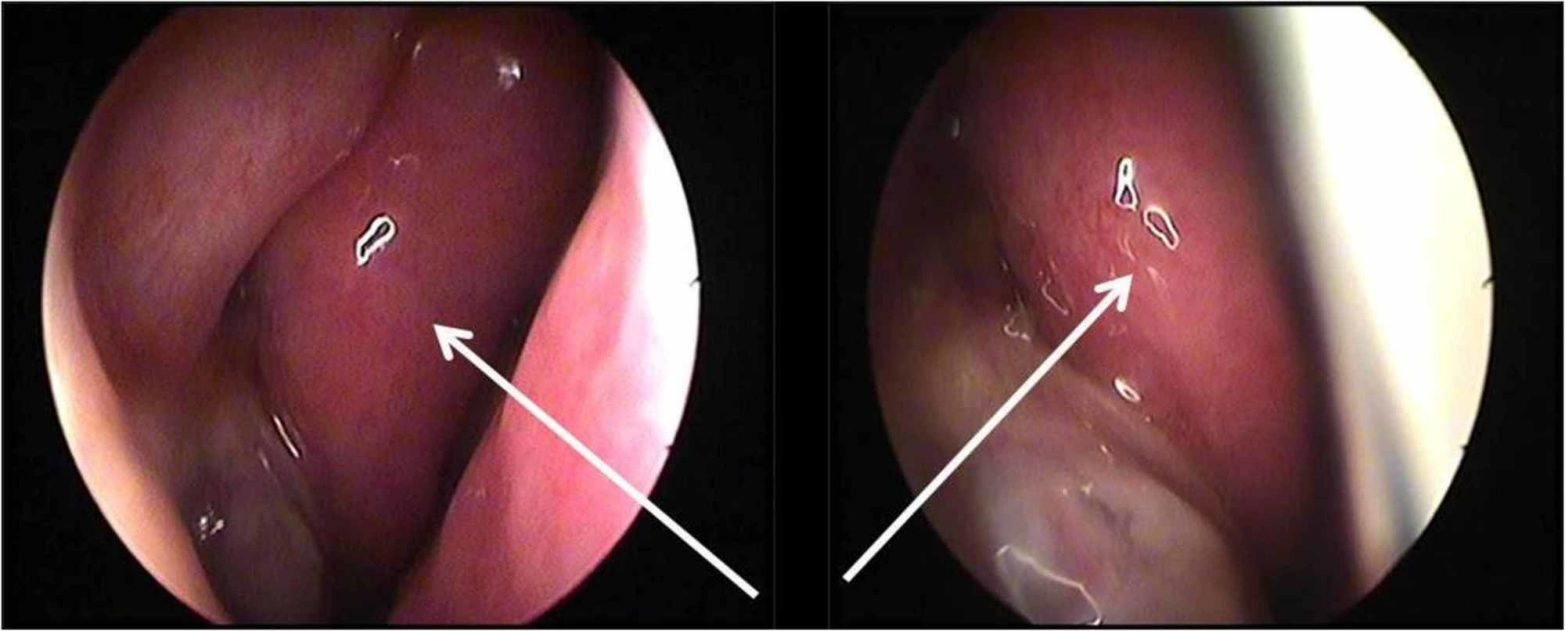
Imaging of the lesion in the right nasal cavity, middle meatus

A 74-year-old woman was referred to the Otolaryngology, Head and Neck Surgery Department with complaints of obstruction on the right nasal cavity. After thorough history taking, she would reveal frequent mild nosebleeds and a mild feeling of pressure in the right side of her face during the past year. As far as her medical history is concerned, she reported hypertension and Behcet's syndrome under treatment. She reported no known allergies and no smoking or alcohol drinking habits. No relevant past interventions were mentioned. Endoscopy revealed a mass lesion adhered to the middle meatus causing complete obstruction of the right nasal cavity (Figure 1). 

**Figure 2:**
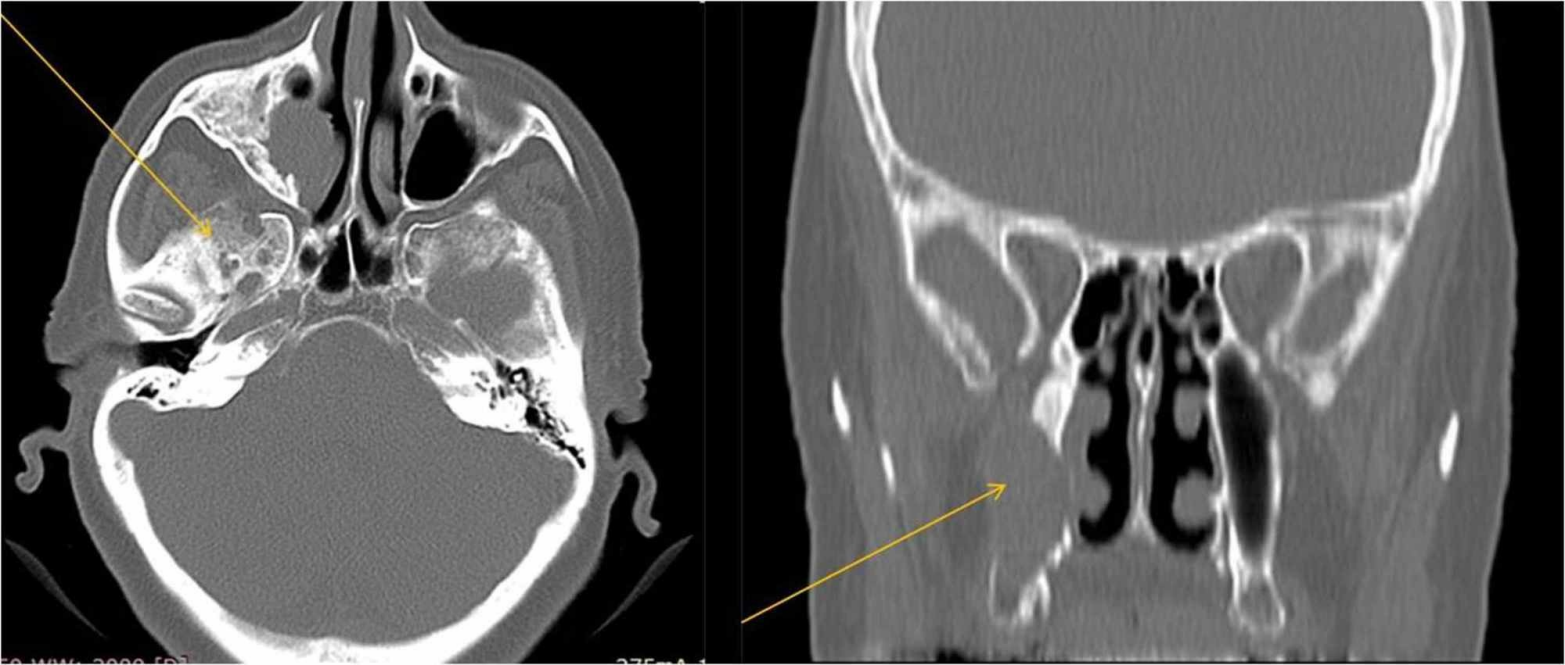
CT imaging of the paranasal sinuses, revealing a soft tissue lesion occupying the maxillary sinus and nasal cavity of the right side with disintegration of the outside and inside wall

Computed tomography of the paranasal sinuses revealed a soft tissue density lesion occupying the maxillary sinus and nasal cavity of the right side. This measured 2.6 cm x 3.1 cm x 3 cm and extended to the right middle and inferior meatus of the right nasal cavity. Disintegration of the outside and inside wall on the maxillary sinus was also obvious (Figure 2). Magnetic resonance imaging added no further relevant information. Biopsy of the lesion with local anaesthesia indicated benign epithelial odontogenic tumour.

**Figure 3:**
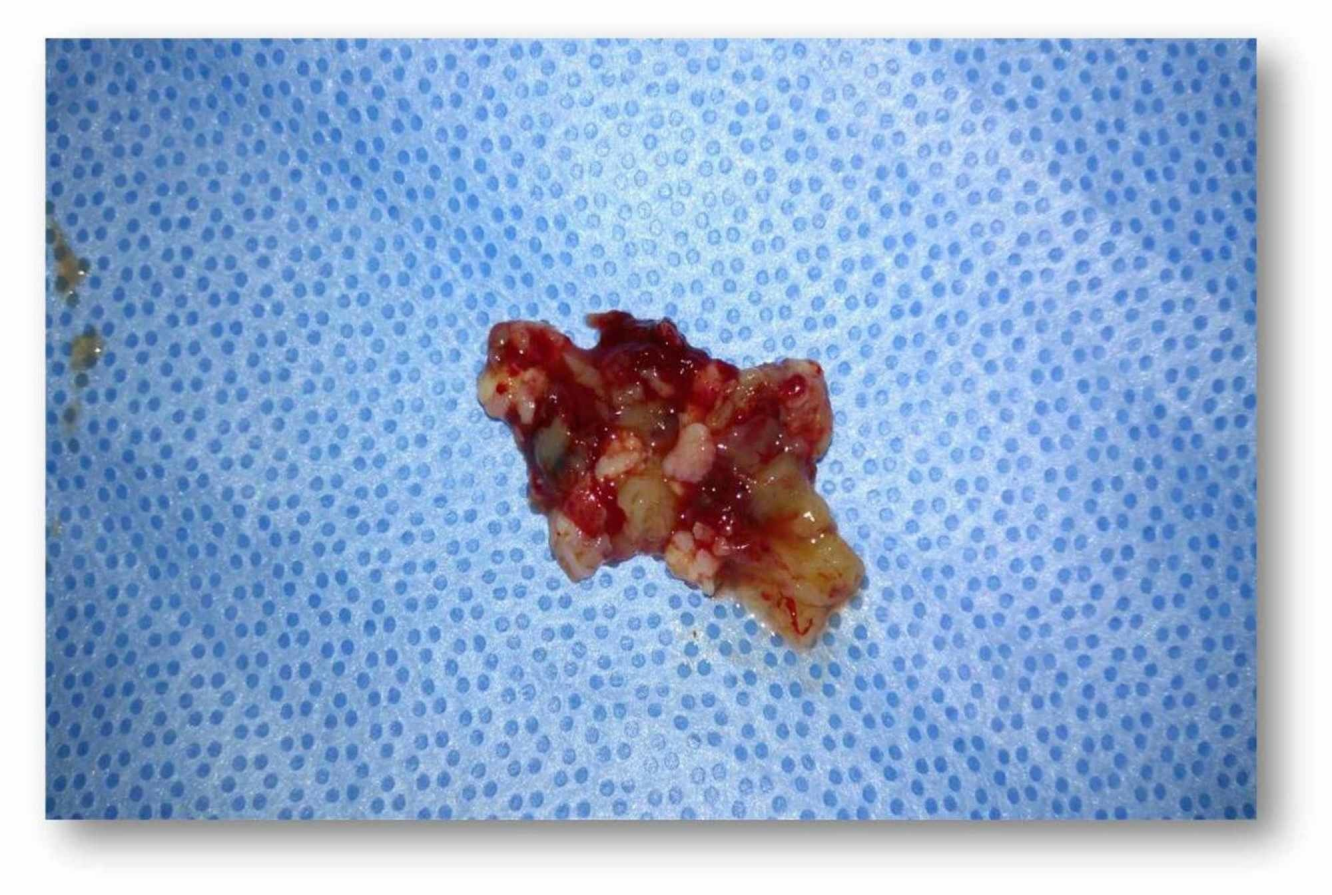
En bloc resection of the solid/multicystic tumour from the maxillary sinus

Treatment with surgical removal of the lesion was decided. Endoscopic sinus operation was performed under general anaesthesia. The patient was subjected to maxillary sinus opening and removal of the inner wall. The lesion was resected en bloc from the inferior and anterior wall. Part of the periosteum was also removed from the maxillary sinus. The sample was sent for histological study (Figure 3). The biopsy findings confirmed follicular ameloblastoma (solid/multicystic).

**Figure 4:**
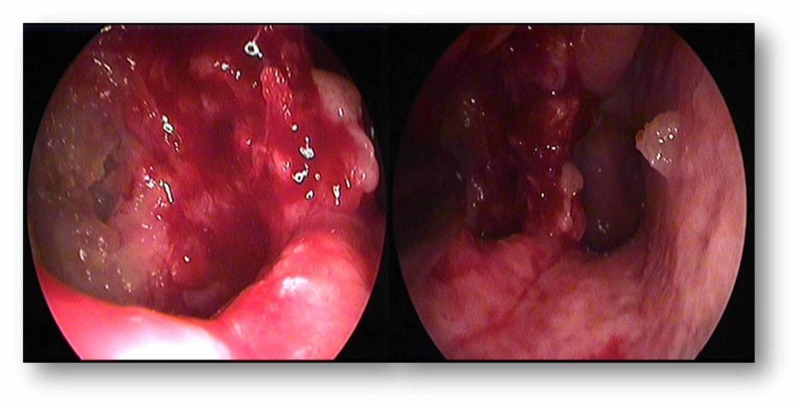
Endoscopic endonasal depiction of the internal surface of the maxillary sinus revealing free airflow three months postoperatively

Postoperatively, no complications were reported and the patient was discharged three days later. Ten days postoperatively, endoscopy revealed unobstructed airflow through the right nasal cavity (Figure 4). Three months of follow-up unveiled no complication and symptoms were effectively alleviated.

## Discussion

Ameloblastoma was initially recognized from Cusak as a neoplastic proliferation of the mandible in the early 19th century, but a more detailed description came 50 years later by Falckson [6]. Τhis kind of tumour is small, hard, frequently lobed, characterized by the presence of cysts and the deposition of calcium salts on its inner mass. Depending on what form of structure prevails, it can be discerned as solid or cystic. Histologically, ameloblastomas have been differentiated into 10 types and it is possible that two or more types coexist in the same lesion. The follicular and plexiform types form two-third of the ameloblastomas [1,3]. 

Ameloblastoma is known to grow slowly and asymptomatically [4]. It grows at the expense of surrounding tissues and if it is greatly increased in size, it is likely to end in facial deformity [2-4]. In neglected cases, severe abnormalities of the face and jaw have been observed [2]. If an aggressive tumour is left without treatment, it can obstruct the nasal and oral airways, making it impossible to breathe without oropharyngeal intervention [4,5]. In our case, right nasal obstruction and disintegration of the outside and inside wall on the maxillary sinus was observed. Insignificant symptoms and unusual location, as in our instance, usually lead to delay in diagnosis and treatment [4].

Wide resection of the process into healthy tissues, safe margins, and immediate reconstruction whenever necessary and possible is the treatment of choice [2,7]. Local relapse is rare [2]. Routine histological classification of the ameloblastoma is mandatory for its morphological characterization and, thus, a better treatment delineation [3]. The main factors associated with successful treatment are early diagnosis and the correlation of histopathologic findings with clinical and imaging features to achieve a correct definitive diagnosis. In this kind of lesions, preoperative diagnosis may alter the therapeutic decision and finally lead to prognostically different biologic behaviour of the tumour [4,5,7].

## Conclusions

Ameloblastoma is a rare, mostly benign tumour derived from dental mesenchyme. Insignificant symptoms and unusual location commonly lead to delay in diagnosis and treatment. Preoperative diagnosis is critical and may alter the therapeutic management. Choice of treatment may lead to prognostically different biologic behaviour of the tumour.

## References

[1] Bredenkamp JK, Zimmerman MC, Mickel RA (1989). Maxillary ameloblastoma. A potentially lethal neoplasm. Arch Otolaryngol Head Neck Surg.

[2] Sarnat BG, Bradley JP (2010). Craniofacial Biology and Craniofacial Surgery.

[3] Batsakis JG, McClatchey KD (1983). Ameloblastoma of the maxilla and peripheral ameloblastomas. Ann Otol Rhinol Laryngol.

[4] Kahn MA (1989). Ameloblastoma in young persons: a clinicopathologic analysis and etiologic investigation. Oral Surg Oral Med Oral Pathol.

[5] Gardner DG, Pecak AM (1980). The treatment of ameloblastoma based on pathologic and anatomic principles. Cancer.

[6] Masthan KM, Anitha N, Krupaa J, Manikkam S (2015). Ameloblastoma. J Pharm Bioallied Sci.

[7] Faisal M, Abbas T, Khaleeq U (2017). Treatment outcomes of rare retromolar trigone squamous cell carcinoma using combined modalities. Cureus.

